# Effect of Conventional and Game-based Teaching on Oral Health Status of Children: A Randomized Controlled Trial

**DOI:** 10.5005/jp-journals-10005-1297

**Published:** 2015-08-11

**Authors:** Yogesh Kumar, Sharath Asokan, Baby John, Thiruvenkadam Gopalan

**Affiliations:** Postgraduate Student, Department of Pedodontics, KSR Institute of Dental Science and Research, Tiruchengode, Tamil Nadu, India; Reader, Department of Pedodontics, KSR Institute of Dental Science and Research, Tiruchengode, Tamil Nadu, India; Professor and Head, Department of Pedodontics, KSR Institute of Dental Science and Research, Tiruchengode, Tamil Nadu, India; Postgraduate Student, Department of Pedodontics, KSR Institute of Dental Science and Research, Tiruchengode, Tamil Nadu, India

**Keywords:** Flash cards, Oral health education, Playway method.

## Abstract

**Aim:** To compare the effectiveness of conventional and game-based teaching on the level of knowledge and practice regarding oral hygiene among 7 to 10-year-old school children.

**Materials and methods:** A total of 60 children aged 8 to 10 years were randomly divided into two groups: groups A and B. The intervention was started after the pretest evaluation of their knowledge regarding oral health and estimation of Debris Index-simplified (DI-S). Children in group A were given oral health education through flash cards once daily for 7 days. Children in group B were educated through the play-way method, i.e. connect the dots game combined with flash cards. The evaluations, regarding oral hygiene and DI-S were recorded on the 8th day after intervention. A follow-up score was also recorded after 1 and 3 months. Statistical analysis was done using paired t-test and Chi-square test.

**Results:** There was significant increase in oral hygiene scores and decrease in debris scores compared to baseline in both groups at 1 week and 1 month. At 3 months interval, both groups showed a decrease in oral hygiene scores from baseline with group B showing highly significant reduction. The mean increase in knowledge score was also significantly better in group B (p < 0.05).

**Conclusion:** The connect the dots game that includes oral health guidelines including good dental hygiene and dietary habits can thus be an effective intervention aid for teaching the basic oral health concepts among school going children.

**How to cite this article:** Kumar Y, Asokan S, John B, Gopalan T. Effect of Conventional and Game-based Teaching on Oral Health Status of Children: A Randomized Controlled Trial. Int J Clin Pediatr Dent 2015;8(2):123-126.

## INTRODUCTION

Dental caries is a global disease with widespread prevalence, particularly among children. One of the reasons for this is due to increased availability of processed foods and beverages, which contain refined sugars.^1^ Oral health is an integral part of general health and its maintenance is of prime importance. Health education is an important tool in educating school children about the prevention of oral health related problems. The education imparted must inspire the recipient’s mind and not just fill their heads with facts. Education is a threefold process of imparting knowledge, developing skills and interests, attitudes and life values.^2^ One of the ways of achieving the above goals is to integrate education and entertainment, thus making the process of learning an enjoyable one.^3^ Playing games has many benefits for children: it develops their visual alertness, increases their attention span and also assists with memory strategies and reasoning.^4^ A variety of cost effective media and materials are readily available and can be used to make learning both interesting and effective. Game-based teaching is an educational strategy that facilitates and reinforces child’s learning in a stimulating and dynamic format. It can be an alternative for teaching basic health concepts.^5^ Various methods like board games,^5^ drama,^6^ robots^7^ have been used for health education purposes. There is limited evidence regarding the effectiveness of games in oral health education and the resultant change in oral hygiene status.

The present study, the first of its kind, was done to compare the efficacy of conventional (flash cards) and game based (connect the dots) teaching on oral health status among 7 to 10-year-old children.

## MATERIALS AND METHODS

The study protocol was analyzed and approved by the Institutional Review Board. Written consent was obtained from the parents of all the participating children. A total of 60 school going children between 7 and 10 years of age participated in the study. Each group of thirty children was randomly assigned into two groups: group A―educated with conventional (flash card) method; group B―educated with both flash card and game based (connect the dots) method. Randomization pattern was obtained through computer generated software. A self administered closed ended questionnaire was given to children to assess their baseline level of knowledge regarding oral hygiene. The questionnaire consisted of demographic data and 11 questions regarding oral health, brushing and diet. The questions were read out and also explained in the local language to eliminate bias. Each question was scored as 0 (wrong answer) or 1 (correct answer) and hence the total score ranged from 0 to 11. The Debris Index-simplified (DI-S) was estimated on the six index teeth and was recorded. The score ranged from 0 to 3. The intervention was started after the pretest evaluation of their knowledge regarding oral health and estimation of DI-S.

**Fig. 1:**
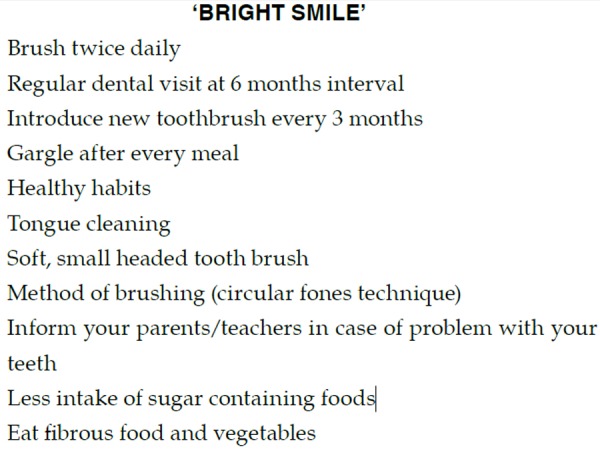
Bright smile anagram

**Fig. 2:**
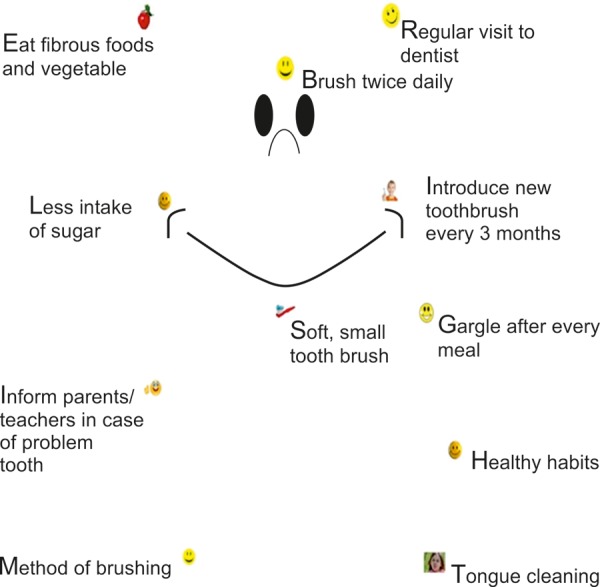
Connect the dots game

For the purpose of educating the children we developed an anagram and connect the dots game. The anagram used was ‘Bright Smile’ (Fig. 1), where each alphabet represented a specific oral hygiene instruction. Connect the dots game (Fig. 2) was developed in the form of tooth structure with 11 alphabets as in the anagram and oral hygiene instruction.

In group A, the children were shown the flash cards with a picture in front and oral hygiene instructions behind. Whereas in the group B, apart from the flash card education, the children were engaged in ‘connect the dots’ game. Children were told to connect the dots between two alphabets and read out the oral hygiene instructions written along with it. When the dots were connected children could see a happy ‘Bright Smile’ tooth. This helped the children realize that following the set of instructions would give them a bright smile. This intervention was continued once daily for a period of 1 week. The evaluations, regarding oral hygiene and DI-S were recorded on the 8th day after intervention. A follow-up score was also recorded after 1 and 3 months.

Statistical analysis of data was done using SPSS software (15.0, SPSS Inc., Chicago Ill, USA). Paired ‘t’ test and Chi-square test were used to compare the quantitative and qualitative variables respectively. p < 0.05 was considered significant.

## RESULTS

The attrition was 10% (n = 3) in group A and 6.5% (n = 2) in group B. Table 1 shows that there was a significant decrease in Debris index score from baseline to post 3 months data in both groups A and B. However, the difference in group B was highly significant. On categorizing, the Debris index scores, it was seen that there was a significant increase from 0 to 15 children with good oral hygiene in group B compared to group A (Table 2). In group B, the knowledge score showed an increase in mean percentage difference by 28.1% at 3 months follow-up when compared to baseline values. In group A, the knowledge score increased significantly at 1 week interval; showed a gradual decrease over the follow-up period and nearly reached the baseline values. At the end of 3 months, there was 3.9% increase in mean percentage difference in group A (Table 3). Both the groups followed a similar trend in debris index scores and knowledge score from baseline to 3 months. Initially, there was a highly significant increase in the scores immediately after intervention which gradually decreased over the follow-up period.

## DISCUSSION

The history of dentistry has established the fact that prevention through oral health education is a major factor in controlling dental caries and related complications. Childhood is a significant time for intellectual growth and personality development. Young children are particularly receptive during this phase of growth.^8^ Equally important is the need to understand differences in the mental cognitive ability of the children at different ages and the need to develop different intervention programs for different age groups. Castillo et al (2001) conducted an interventional study to determine the effectiveness of an educational strategy based on Children’s games (snake and ladder) for teaching the basic health concepts to school-age children and concluded that using games that include health and hygiene messages can be an alternative for teaching basic health concepts.^5^ A study was conducted to assess the effectiveness of the snake and ladder game on knowledge of common ailments among school children and the study revealed that there was a significant difference in the knowledge scores after administrating the intervention.^9^ This game was originally used by religious leaders to teach children about the difference between good and evil-climbing up the ladders representing good, and sliding down the snakes representing evil.^10^ John et al showed that drama can be used for better impact on oral health attitude and practices in preschool children.^6^ Ahire et al described a technique in which a robot (ROBOTUTOR) was used to demonstrate Bass toothbrushing technique to adults and concluded that it can save clinician’s chair side time as well as help in effective demonstration of the brushing technique.^7^ The present study is the first to put forth the oral health intervention concept and also the reinforcement of messages on oral health through the play-way method.

**Table 1:** **Table 1:** Comparison of mean Debris index score between two groups

*Group/timeline*		*Baseline*		*Post 1 week*		*Post 1 month*		*Post 3 months*		*p-value (baseline to 3 months)*	
A Flash cards		1.26 ± 0.37		0.74 ± 0.27		0.82 ± 0.38		0.94 ± 0.34		< 0.01	
B Flash cards + game		1.30 ± 0.35		0.68 ± 0.31		0.69 ± 0.3		0.73 ± 0.25		< 0.001	

**Table 2:** **Table 2:** Comparison of Debris index inference between the two groups

				*Baseline*		*Post 1 week*		*Post 1 month*		*Post 3 months*	
Group A		Good		3		17		14		7	
Flash cards		Fair		25		12		14		20	
		Poor		2		0		1		0	
Group B		Good		0		20		18		15	
Flash cards + game		Fair		26		8		10		13	
		Poor		4		0		0		0	

**Table 3:** **Table 3:** Comparison of mean knowledge score between the two groups

*Group/timeline*		*Baseline*		*Post 1 week*		*Post 1 month*		*Post 3 months*		*Mean increase (%)*		*p-value*	
A		8.43 ± 1.22		9.93 ± 0.99		9.45 ± 1.43		8.76 ± 1.41		3.9		NS	
Flash cards													
B		7.20 ± 2.14		9.36 ± 1.66		9.29 ± 1.44		9.07 ± 1.54		28.21		0.046	
Flash cards + game													

NS: Non significant

Games can be used as an innovative and challenging educational tool. They have long been used as a teaching strategy in both child and adult education, promoting self-learning and participation. By involving repetition and allowing important points to be reiterated, games appear to increase retention and application.^4^

In the present study, connect the dots game was developed for educating the children. In cognitive psychology, connecting the dots test (trail making test) was used to provide information about visual search speed, scanning, speed of processing, mental flexibility, as well as executive functioning. It consists of two parts in which the subject is instructed to connect a set of 25 dots as fast as possible while still maintaining accuracy.^11^ The auditory and visual working memory performance in children improves with age. Visual working memory reaches functional maturity earlier than the corresponding auditory system. Hence, younger children rely on visual codes to remember.^12^ In the present study, a game based intervention program that relied on visual coding increased the visual alertness among children in group B that helped them understand oral health instructions easily. The instructions were remembered well and also retained for a longer period of time in children belonging to group B. This was evident by a significant increase in the knowledge scores (28.1%) and decrease in the debris scores calculated immediately after the intervention program. The significant difference in scores after 1 week may be due to Hawthorne effect where individuals may change their behavior due to the attention they received from researchers. In the study by Castillo et al, the data were recorded immediately after the intervention, whereas in the present study, a follow-up data of 3 months were obtained to improve the reliability. These follow-up scores remained above the baseline value in both the groups. The improvement in control group may be attributed to the various other factors such as the ripple effect (information from participants in the other groups), personal home oral hygiene practices, attitude and education status of the parents which were not considered in the study.

The children in the present study belonged to concrete operational period according to Jean Piaget’s cognitive theory. The concrete operational stage is signified by the child’s ability to master the conservation tasks due to their growing interest in participation of various activities. The conservation task involves exposure to situations that require the ability to mentally manipulate objects. Children who are capable of mastering the conservation task are able to progress into the fourth stage of development, which involves the ability to think abstractly. The child of this age group belonged to 2nd stage of moral development that is, moral realism. Children at this stage understand the concept of rules, but still see them as external and rigid. They evaluate wrong doing in terms of its consequences, not intentions, and obey rules mainly because they are there. They recognize the sanctity of rules and that they have to play by them and cannot make up new ones to a game.^13^ This helps the children to retain the oral hygiene instructions when given repeatedly and also apply it.

A possible limitation is the limited follow-up period of 3 months which may not allow maturation of dental health messages.^14^ Within the limitations of this pilot trial, we can conclude that connect the dots game with oral health instructions can thus be an effective intervention aid for teaching the basic oral health concepts among younger children. The play-way method’s greater impact and response, cost effectiveness and ease of implementation, justifies that it may be routinely used for providing oral health education to school going children, thus helping in prevention of dental caries.
